# *Shewanella putrefaciens*, a rare human pathogen: A review from a clinical perspective

**DOI:** 10.3389/fcimb.2022.1033639

**Published:** 2023-02-02

**Authors:** Stephanie Müller, Simone von Bonin, Ralph Schneider, Martin Krüger, Susanne Quick, Percy Schröttner

**Affiliations:** ^1^ Department of Medicine I, University Hospital Carl Gustav Carus, Technische Universität Dresden, Dresden, Germany; ^2^ Department of Medicine III, University Hospital Carl Gustav Carus, Technische Universität Dresden, Dresden, Germany; ^3^ Institute for Medical Microbiology and Virology, University Hospital Carl Gustav Carus, Dresden, Germany

**Keywords:** *Shewanella putrefaciens*, human infection, rare bacterial pathogen, Shewanella, rare human pathogen

## Abstract

*Shewanella putrefaciens* is a gramnegative, facultatively anaerobic, rod shaped bacterium. It belongs to the class of the *Gammaproteobacteria* and was first described in 1931. *S. putrefaciens* is part of the marine microflora and especially present in moderate and warm climates. The bacterium is a rare oppurtonistic human pathogen associated mainly with intra-abdominal as well as skin and soft tissue infections. However, it has also been reported in association with more severe diseases such as pneumonia, intracerebral and ocular infections and endocarditis. In these cases the clinical courses are often associated with underlying, predisposing diseases and risk factors. For successful treatment of *S. putrefaciens*, a combination of appropriate local therapy, e.g. surgical treatment or drainage, and antibiotic therapy should be performed. Since multiple resistances to antibiotics are described, the results of the antimicrobial susceptibility testing must be considered for effective therapy as well. Furthermore, a main challenge in clinical practice is the accurate microbiological identification, and especially the correct differentiation between *S. putrefaciens* and *S. algae*. Under certain circumstances, *Shewanella*-infections can have severe, sometimes even fatal consequences. Therefore, we decided to present the current state of knowledge as well as further aspects with regard to future diagnostics, therapy and research.

## Introduction

The species *Shewanella putrefaciens* is a gram-negative bacterium first described in 1931 (Derby and Hammer, 1931). It is part of the marine microflora and especially present in moderate and warm climates (Holt et al., 2005; Vignier et al., 2013; López Aperador et al., 2016; Yu et al., 2022). In addition, it is an important spoilage agent of protein-rich refrigerated foods (Brink et al., 1995; Vogel et al., 1997).

*S. putrefaciens* is a rare oppurtonistic human pathogen (Tsai et al., 2008). The bacterium is mainly associated with skin- and soft-tissue and intra-abdominal infections, especially biliary tract infections and peritonitis (Chen et al., 1997; Holt et al., 2005; Vignier et al., 2013). *S. putrefaciens* can also lead to bacteremia with septic and possibly even lethal courses (Brink et al., 1995; Chen et al., 1997; Tang et al., 2016). *S. putrefaciens* infections are often polymicrobial, so the human pathogenic role of the bacterium requires further clarification (Brink et al., 1995; Chen et al., 1997; Yu et al., 2022).

According to the ”List of Prokaryotic names with Standing in Nomenclature” (LSPN) there are currently 80 species of the genus Shewanella validly described (Parte et al., 2020). To the best of our knowledge, only 3 human pathogenic Shewanella spp. have been described so far. Thus, *S. putrefaciens*, *S. algae* and *S. xiamenesis* have been cultured from clinical isolates (Zong, 2011; Yu et al., 2022). *S. haliotis* has been described in the past as another human pathogenic Shewanella spp. (Poovorawan et al., 2013; Byun et al., 2017). However, Szeinbaum et al., 2018 stated that *S. haliotis* must be identified as *S. algae* (Szeinbaum et al., 2018). Previously, *S. algae* was considered a subspecies of *S. putrefaciens* (Khashe and Janda, 1998; Tsai et al., 2008). It was not until 1990 that *S. algae* was described by Simidu et al., 1990 as a new species of the genus Shewanella (Simidu et al., 1990; Nozue et al., 1992). In the following years, subsequent studies and investigations revealed that probably more than 80% of human infections previously thought to be caused by *S. putrefaciens* are actually caused by *S. algae* (Holt et al., 2005; Tsai et al., 2008). This is due to the fact that biochemical and phenotypic characterization tests, respectively, and conventional bacterial identification systems are hardly able to correctly distinguish between these two clinically relevant Shewanella species (Tsai et al., 2008; Sharma and Kalawat, 2010; Vignier et al., 2013; Benaissa et al., 2021). Therefore, currently correct pathogen identification requires analyses beyond routine daily diagnostics.

In this review, we summarize the presently available knowledge of *S. putrefaciens* infections and describe the microbiological aspects of the species, the difficulties of species identification, pathogenicity, clinical features, the antimicrobial susceptibility, options for antimicrobial treatment and aspects of further research.

## Search strategy and selection of articles

A literature search in PubMed was performed using the following keywords: `*Shewanella putrefaciens* AND Infection´. All studies published in PubMed since the initial description up to March 31^st^ 2022 were included. All available manuscripts related to *S. putrefaciens* and references cited in the relevant articles were evaluated for their relevance for the topic of this review. Only case reports of human infections caused or associated with *S. putrefaciens* were included. *S. algae* was frequently misclassified as *S. putrefaciens* in the past and was first described only in 1990, so published case reports attributed to *Achromobacter putrefaciens* or *Pseudomonas putrefaciens* were excluded (Simidu et al., 1990; Nozue et al., 1992; Vogel et al., 1997; Holt et al., 2005; Tsai et al., 2008; Vignier et al., 2013).

## History and taxonomy

*S. putrefaciens* was first described by Derby and Hammer in 1931 (Derby and Hammer, 1931). They isolated a strain from putrified butter, which was initially identified as *Achromobacter putrefaciens.* Further studies have shown that it is a species that has not yet been described (Nozue et al., 1992; Holt et al., 2005). In 1941, it was assigned to the genus *Pseudomonas* and subsequently named *Pseudomonas putrefaciens* (Long and Hammer, 1941). According to Shewan et al., 1960 in the following decades this organism was classified in Pseudomonas group IV (Shewan et al., 1960). Based on the G+C content, the assignment to the genus of *Alteromonas* was made in 1972 (Baumann et al., 1972). However, based on phylogenetic studies, these organisms were reclassified in 1985 to the new genus *Shewanella*, named after the scotish bacteriologist James M. Shewan (Chaudhary et al., 2022), and included in the family *Vibrionaceae* (MacDonell and Colwell, 1985; Holt et al., 2005). In 1990, Simidu et al. isolated a new mesophilic Shewanella species from red alga and described it as *S. alga* (Simidu et al., 1990; Nozue et al., 1992; Vogel et al., 1997). Nozue et al. concluded in 1992 that the vast majority of strains previously identified as *S. putrefaciens* should be categorized as *S. alga* (Nozue et al., 1992). Finally, in 2004, Ivanova et al. introduced the familiy *Shewanellaceae* with *Shewanella* as the type genus (Ivanova et al., 2004). Today, according to the ”List of Prokaryotic names with Standing in Nomenclature” (LPSN), *S. putrefaciens* belongs to the family *Shewanellaceae* within the class of *Gammaproteobacteria* (Ivanova et al., 2004; Parte et al., 2020). As far as we know, *S. putrefaciens* is one of 3 human pathogenic Shewanella spp. known to date (Zong, 2011; Yu et al., 2022).

## Phenotypic characteristics of *Shewanella putrefaciens*

*S. putrefaciens* is a gramnegative, facultatively anaerobic, rod shaped, oxidase and catalase positive, motile bacterium with a single polar flagellum (Nozue et al., 1992; Héritier et al., 2004; Holt et al., 2005; Sharma and Kalawat, 2010; Yu et al., 2022). The bacterium has a G+C content between 45-48 mol% and grows in 1-2mm large, yellowish-brown colonies (Nozue et al., 1992; Holt et al., 2005; Jayalekshmi et al., 2022). Hydrogen sulfide generation is the main phenotypic feature (Holt et al., 2005). According to Vogel et al., 1997 various electron acceptors such as trimethylamine N-oxide (TMAO), elemental sulfur, nitrate, iron, thiosulfate, manganese or fumarate can be dissimilatory reduced by this bacterium (Vogel et al., 1997).

As far as we know, *S. algae* and *S. putrefaciens* are two of three potentially human pathogenic Shewanella-species isolated from clinical specimens to date (Zong, 2011; Vignier et al., 2013; Benaissa et al., 2021; Yu et al., 2022). After the initial description of the species *S. putrefaciens*, *S. algae* were often misidentified as *S. putrefaciens* (Sharma and Kalawat, 2010). A strain that parasitizes in red algae was first described as *S. alga* in 1990 by Simidu et al. (Simidu et al., 1990; Nozue et al., 1992; Vogel et al., 1997; Pagniez and Berche, 2005). Compared to *S. algae*, *S. putrefaciens* has stronger saccharolytic activity (Holt et al., 2005). According to Holt et al., 2005, the bacterium is able to produce acid from maltose, glucose, partially also from sucrose and arabinose, while *S. algae* usually metabolizes only ribose (Holt et al., 2005). In addition, unlike *S. putrefaciens*, *S. algae* also grows at 42°C, on Salmonella-Shigella agar, has a tolerance to 6% NaCl and forms beta-hemolytic, mucoid colonies on sheep blood agar (Nozue et al., 1992; Holt et al., 2005). However, Vogel et al., 1997 recommended the use of 10% NaCl to distinguish between the two species (Vogel et al., 1997).

## Occurrence and natural habitat

*S. putrefaciens* was first isolated from water supplies of dairies and putrified butter (Derby and Hammer, 1931; Holt et al., 2005).

Their natural occurrence includes all types of water including fresh, marine, river and sewage all over the world (Brink et al., 1995; Chen et al., 1997; Khashe and Janda, 1998; Oh et al., 2008; Vignier et al., 2013; Yu et al., 2022). The bacterium is a component of the marine microflora (Vignier et al., 2013). Geographically, they are mainly found in moderate and warm climates (Holt et al., 2005; López Aperador et al., 2016). In addition, they have already been detected in natural energy reserves such as petroleum brines or natural gas (Chen et al., 1997; López Aperador et al., 2016).

They have also been isolated from a variety of foods including milk, cream, butter, eggs, poultry, (raw) fish or seafood and beef products (Chen et al., 1997; Khashe and Janda, 1998; Bulut et al., 2004; Oh et al., 2008). *S. putrefaciens* is a biofilm former and able to reduce TMAO to trimethylamine (Vogel et al., 1997; Bagge et al., 2001; Holt et al., 2005; Jayalekshmi et al., 2022). This seems to be a relevant reason why this bacterium is an important spoilage agent of protein-rich refrigerated foods, especially for frozen white-fleshed fish from temperate waters (Brink et al., 1995; Vogel et al., 1997; Jayalekshmi et al., 2022).

## Identification of *Shewanella putrefaciens* in routine diagnostics

Proper differentiation between *S. putrefaciens* and *S. algae* in daily routine diagnostics is challenging. Both Shewanella spp. grow after an incubation period of 18-24 hours on conventional solid culture media, *S. putrefaciens* e.g. on Luria-Bertani (LB) broth, and on media commonly used in microbiological diagnostics, such as Mac-Conkey agar (Holt et al., 2005; Jayalekshmi et al., 2022).

In a study published in 1992, Nozue et al. found that *S. putrefaciens* strains with a high G+C content of 52 to 54 mol% did not belong to the type strain of *S. putrefaciens* (ATCC 8071) but to that of *S. alga*, later referred to as *S. algae* (Nozue et al., 1992; Trüper and De’Clari, 1997; Khashe and Janda, 1998). Recent data indicate that more than 80% of clinical *S. putrefaciens* isolates have been misidentified in the past and probably need to be assigned to *S. algae* (Holt et al., 2005; Tsai et al., 2008; Vignier et al., 2013). This is due to the fact that both conventional bacterial identification systems and biochemical testing methods cannot clearly distinguish between the both (Tsai et al., 2008; Sharma and Kalawat, 2010; Vignier et al., 2013; Benaissa et al., 2021). For example, the databases of semi-automatic and automatic systems such as Vitek 2, API ID 32 GN, API 20E and 20 NE contain *S. putrefaciens* but not *S. algae*, which may lead to misidentification as *S. putrefaciens* due to the high similarity of both species (Holt et al., 2005; Vignier et al., 2013; Yu et al., 2022). By performing 16S rRNA analyses on three isolates, previously identified as *S. putrefaciens* by biochemical assays, Vignier et al., 2013 were able to correctly identify all three isolates as *S. algae* by molecular analyses (Vignier et al., 2013). To our knowledge, MALDI-TOF mass spectrometry also appears to be a good method for identifying Shewanella spp. but requires further analysis (Byun et al., 2017; Yu et al., 2021; Yu et al., 2022). Therefore, analysis beyond routine daily diagnostics by 16S rRNA sequencing, ribotyping, or whole-cell protein profiling is required to correctly distinguish between the two Shewanella spp. (Vogel et al., 1997). In our experience, whole genome sequencing followed by digital DNA-DNA hybridisation (dDHH) has proven to be very useful in determining the actual species present (Kopf et al., 2021). This procedure is nowadays regarded as the gold standard of molecular species identification. It can therefore be assumed that *S. putrefaciens* and *S. algae* can also be sufficiently identified using this method (Richter and Rosselló-Móra, 2009). Futhermore, a more advanced method to distinguish between individual Shewanella spp. is multilocus sequence analysis (MLSA) of different protein-coding genes (Fang et al., 2019).

## Pathogenicity and potential virulence factors

*S. algae* seems to cause more human infections than *S. putrefaciens* (Nozue et al., 1992; Vogel et al., 1997; Khashe and Janda, 1998; Sharma and Kalawat, 2010). The causative pathomechanisms have not been fully clarified. Based on current knowledge, in human pathogenic Shewanella-subspecies appear to colonize appropriate tissues and subsequently cause local and eventually invasive infection in patients with predisposition (Yohe et al., 1997; Sharma and Kalawat, 2010).

Hepatobiliary disorders, such as cholelithiasis or liver cirrhosis, are important risk factors for *S. putrefaciens* infections (Chen et al., 1997; Yu et al., 2022). In addition, numerous infections of the biliary tract system by this pathogen have been described (Chen et al., 1997). The species has also been isolated in the past from oil, petroleum or fatty foods, so its lipophilia seems to be a possible explanation for its biliary affinity (Chen et al., 1997; Oh et al., 2008). Another important virulence factor appears to be the production of extracellular enzymes such as lecithinase, lipase and DNase (Papanaoum et al., 1998). Local enzyme production favors the development of necrosis of the skin and subcutaneous tissue and thus the development of skin and soft tissue infections (Papanaoum et al., 1998). In addition, *S. putrefaciens* is a biofilm former (Bagge et al., 2001; Holt et al., 2005; Jayalekshmi et al., 2022). This ability could play an important role in causing catheter-associated infections as described by Shrishrimal in 2012 (Shrishrimal, 2012). In addition, *S. putrefaciens* is able to attach to and invade human intestinal epithel cells (Dias et al., 2019). In our view, the causative virulence factors of the bacterium that lead to infections in humans have not yet been fully elucidated.

## Clinical features and risc factors for *Shewanella putrefaciens* infections

Case reports deemed clinically relevant to this review have been compiled in **Supplementary Table 1**.

We identified a total of 87 relevant, published cases of *S. putrefaciens* infections. In 27 cases, it was not possible to make a statement about the sex due to missing information in the individual case reports. The remaining 60 cases were 73.3% male and 26.7% female. Considering only the infections in adults, it was not possible to make a statement about the sex of a total of 7 cases. The remaining 58 cases were 75,9% male and 24,1% female.

Predominantly, the bacterium is associated with skin and soft-tissue infections (Brink et al., 1995; Chen et al., 1997; Yohe et al., 1997; Papanaoum et al., 1998; Pagani et al., 2003; Bulut et al., 2004; Otsuka et al., 2007; Sharma and Kalawat, 2010; Prinja et al., 2013; Mohr et al., 2016; Ryan et al., 2018; Latif et al., 2019; Patel et al., 2020). Under certain circumstances, these can take fatal courses up to necrotizing fasciitis (Yim et al., 2010; Giroux et al., 2017) and the development of a Fournier’s gangrene (Tang et al., 2016). In addition, *S. putrefaciens* can cause arthritides and osteomyelitides (Levy and Tessier, 1998; Carlson and Dux, 2013; Guinetti-Ortiz et al., 2016). Potential entry ports and a typical predisposing factors for corresponding infections are chronic ulcers, especially of the lower extremities, and/or traumatic injuries associated with (sea) water or fish (water) exposure (Oh et al., 2008; Carlson and Dux, 2013; Vignier et al., 2013; Guinetti-Ortiz et al., 2016).

Another major risk factor for infection with this pathogen seems to be an end-stage renal disease. Several cases, particularly CAPD-associated *S. putrefaciens* infections with concomitant peritonitis and, in some cases, associated bloodstream infections, have been published (Chen et al., 1997; Bhandari et al., 2000; Chang et al., 2005; Yim et al., 2010; Shrishrimal, 2012; Lee et al., 2016; López Aperador et al., 2016). *S. putrefaciens* is a biofilm former (Bagge et al., 2001; Holt et al., 2005; Vickers and Ullian, 2011; Jayalekshmi et al., 2022). Therefore, we conclude that dialysis catheters placed in the body, i.e., peritoneal or hemodialysis catheters, are important risk factors and potential entry ports.

Due to the lipophilia of the bacterium, diseases of the bile ducts and also the use of external hepatobiliary drainage catheters are another predisposing factors for *S. putrefaciens* infections (Chen et al., 1997; Oh et al., 2008). Biliary tract infections caused by this Shewanella-species have sometimes been described in association with liver abscess formation (Chen et al., 1997).

Due to traumatic lesions, the pathogen can also lead to severe infections of the eye (Butt et al., 1997; Mohan et al., 2014). Chronic otitis media also appears to be a possible entry port for intracerebral infections (Süzüku et al., 2004; Yilmaz et al., 2007). In the rare case of colonization of the upper and possibly lower respiratory tract by this bacterium, it can also cause severe pneumonia, sometimes accompanied by respiratory failure and the need for ventilation (Holt et al., 2005; Basir et al., 2012; Durdu et al., 2012; Patel et al., 2012).

In the case series published by Brink et al. in 1995, numerous neonatal and pediatric *S. putrefaciens* infections were reported. In particular, low birth weight in combination with poor living standards, especially in premature infants, appears to be a relevant risk factor for bacteremia and, especially, septic or even lethal courses at this age (Brink et al., 1995). Bloodstream infections are not unique to childhood. After all 65.33% (n=75, in 11 cases not reported, in 1 case no blood cultures performed) of the cases in this review were bacteremic. In fact, 2 publications even reported infective endocarditis due to this pathogen, one each with poly- and monomicrobial bacteremia (Dhawan et al., 1998; Constant et al., 2014).

*S. putrefaciens* infections can also cause severe septic courses up to the development of septic shock with multiple organ failure, especially in predisposed individuals. The overall mortality rate in our review was 20% (n=85), but only 18.82% of patients died due to infection. It should be noted that, in addition to the diseases already mentioned, diseases such as diabetes mellitus, peripheral vascular disease, and malignant neoplasms, as well as (drug-induced) immunosuppression, are important risk factors for infection with the described bacterium (Holt et al., 2005; Yilmaz et al., 2007; Basir et al., 2012; Carlson and Dux, 2013; Benaissa et al., 2021). Low socioeconomic status, poor personal hygiene, private or occupational exposure to (sea) water, and consumption of contaminated seafood or fish meat also appear to be predisposing factors for development of *S. putrefaciens* infections (Otsuka et al., 2007; Yilmaz et al., 2007; Oh et al., 2008; Carlson and Dux, 2013; Muñoz et al., 2015).

Most of the Shewanella-infections listed in **Supplementary Table 1** are community-aquired infections. In 2008, Oh et al. reported an in-hospital Shewanella outbreak at a tertiary acute care hospital (Oh et al., 2008). A reused measuring cup for emptying catheter bags was retrospectively identified as the source of the outbreak (Oh et al., 2008). After changing of measuring cups after each use, and adherence to strict hygiene procedures, the local epidemic was contained and controlled (Oh et al., 2008). Shewanella-infections can thus also be spread by contact transmission (Oh et al., 2008).

## Antibiotic susceptibility and treatment

Susceptibility testing is essential, especially regarding targeted anti-infective treatment. Currently, there are no defined criteria for interpreting antibiotic resistance in Shewanella spp. (Yu et al., 2022). Different methods have been used for antimicrobial susceptibility testing. For example, Chen et al., 1997 and Brink et al., 1995 used the disc diffusion method; Otsuka et al., 2007 and Benaissa et al., 2021 used the microdilution method to determine the minimum inhibitory concentration (MIC) (Brink et al., 1995; Chen et al., 1997; Otsuka et al., 2007; Benaissa et al., 2021). Due to the expression of β-lactamases, they often show resistance to penicillin, which is frequently used for the treatment of soft tissue infections (Héritier et al., 2004; Vignier et al., 2013; Ryan et al., 2018). *S. putrefaciens* is usually susceptible to piperacillin, fluoroquinolones, aminoglycosides and carbapenems (Vogel et al., 1997; Holt et al., 2005; Vignier et al., 2013; Muñoz et al., 2015; Ryan et al., 2018; Benaissa et al., 2021). It must be kept in mind that Shewanella spp. may exhibit resistance to imipenem due to possible oxacillinase secretion (Héritier et al., 2004). According to the results of Héritier et al., 2004, *S. algae* KB-1 owns a chromosome-encoded ß-lactamase gene encoding the Ambler class D enzyme OXA-55 (Héritier et al., 2004). OXA-55 usually leads to development of a narrow-spectrum ß-lactam resistance phenotype (Héritier et al., 2004). This oxacillinase has carbapenem-hydrolyzing activity, which explains the lower susceptibility of *S. algae* KB-1 to imipenem (Héritier et al., 2004). There are already reports of infections caused by carbapenem-resistant bacteria (Brink et al., 1995; Otsuka et al., 2007; Baruah and Grover, 2014). So, the use of carbapenems should be avoided.

Variable susceptibility is seen to ampicillin and cephalosporins, with the majority of clinical isolates testing susceptible to third and fourth generation cephalosporins (Chen et al., 1997; Holt et al., 2005; Benaissa et al., 2021; Yu et al., 2022). Interestingly, *S. putrefaciens* is usually susceptible to erythromycin, a macrolide antibiotic, which is particularly effective in the gram-positive range (Chen et al., 1997). An important distinction feature between *S. algae* and *S. putrefaciens* is the susceptibility to polymixin (Holt et al., 2005; Benaissa et al., 2021). Holt et al., 2005 reported that *S. algae* were resistant to colistin, whereas *S. putrefaciens* isolates tested susceptible (Holt et al., 2005).

According to the current literature, infections with *S. putrefaciens* should be treated, also depending on the primary focus, by antibiotic therapy in combination with a sufficient local therapy (Yohe et al., 1997; Bulut et al., 2004; Süzüku et al., 2004; Holt et al., 2005). As part of empiric anti-infective therapy, intravenous treatment with a fluoroquinolone or a betalactam may be considered initially (Holt et al., 2005). Because of the potential for beta-lactamase expression, a combination with a beta-lactam inhibitor should be considered if a beta-lactam is used (Héritier et al., 2004). In severe courses, especially with existing sepsis, intravenous combination treatment using, for example, an aminoglycoside antibiotic should be considered (Brink et al., 1995). Depending on resistance testing and the clinical course, oral sequential antibiotic therapy may be appropriate in special cases (Guinetti-Ortiz et al., 2016; Patel et al., 2020).

## Summary

*S. putrefaciens* is a rare human pathogenic bacterium whose infections can lead to serious clinical or even fatal consequences, especially in predisposed individuals. Microbiological identification and especially correct differentiation between *S. putrefaciens* and *S. algae* is difficult because biochemical and phenotypic characterization tests, respectively, provide insufficient discriminatory criteria and *S. algae* is not included in the databases of most commercial identification systems. Following the change in nomenclature and the distinction between *S. putrefaciens* and *S. algae*, numerous case reports were reexamined in relation to the human pathogen. Recent studies and data suggest that there should be the majority of clinical *S. putrefaciens* isolates assigned to the species *S. algae* (Vogel et al., 1997; Khashe and Janda, 1998; Holt et al., 2005; Tsai et al., 2008; Vignier et al., 2013). Therefore, 16S rRNA sequencing, ribotyping, or whole-cell protein profiling is required to correctly distinguish the two Shewanella spp. (Vogel et al., 1997). On this basis, it can be assumed that the majority of *S. putrefaciens* infections listed in this review are indeed infections caused by *S. algae*. Only in one case report, 16S rDNA amplification assay was performed (Duan et al., 2015). In some case reports, no information was provided on the identification method (**Supplementary Table 1**). Therefore, the present review should be considered under the above limitations.

*S. putrefaciens* can lead to skin and soft tissue infections, arthritides and osteomyelitides, intracerebral, ocular, respiratory and intra-abdominal infections as well as severe bloodstream infections with septic courses and rarely even endocarditis. Severe courses are often associated with underlying, predisposing diseases and other risk factors. *S. putrefaciens* may exhibit intrinisic resistance to penicillins and possibly also to carbepenems. For successful treatment of *S. putrefaciens*, a combination of appropriate local therapy and antibiotic therapy should be performed, taking into account current susceptibility testing.

To prevent nosocomial infections through contact transmission, according to Oh et al., 2008, the following points should be observed in addition to the usual rules of hospital hygiene: wearing gloves during direct patient contact and contact with potentially infectious fluids, washing hands before and after patient care, following strict “no-touch” techniques when draining body fluids, and providing single-use products whenever possible (Oh et al., 2008).

## Author contributions

PS had the idea for this mini-review. PS and SM worked together to develop the concept. SM, PS and MK performed the literature research. SM performed the data analysis and wrote the first draft of the manuscript. All authors reviewed and improved the manuscript and approved the submission.
