# Supplementary material for: Shewanella putrefaciens, a rare human pathogen: A review from a clinical perspective
Source: Front Cell Infect Microbiol. 2023 Feb 2;12:1033639. doi: 10.3389/fcimb.2022.1033639 (PMC9933709; doi:10.3389/fcimb.2022.1033639)
Supplement: Supplementary file 1 [file Table_1.pdf]

Case No.	Reference	Age (Years)/ Sex	Risk facts	Comorbidities	Clinical presentation	Source/ Identification	Potential entry port	Poly- (P)/ Monomi-crobial (M)	Bacteremia	Antibiotic treatment	Outcome
**Skin and Soft-tissue infections**											
**1.**	Bulut et al., 2004	27/M	Sea water exposure	-	Cellulitis of the scalp	Fine needle aspiration from the left parietal scalp/ Biochemical reactions and API ID 32 GN (BioMerieux, Marcy-I'Etoile, France)	Head trauma under the sea	M	Not reported	Cefa; Cipro	Clinical improvement
**2.**	Papanaoum et al., 1998	67/M	Sea water exposure, immuno-suppression	Systemic lupus erythematosus; asthma, multiple squamatosus cell carcinomas of the skin, amputated toe due to osteomyelitis, contained granulomas with acid-fast bacilli on the left arm 2 years ago	Severe cellulitis on the left leg	Swab of the leg ulcers/ API 20NE system (bio Mérieux sa, Lyon, France)	Leg ulcers	M	No	Peni + Genta; Cipro	Clinical improvement
**3.**	Sharma and Kalawat, 2010	57/F	Malignancy, HBD	Breast carcinoma, hepatitis B	Infected ulcer	Not reported/ Biochemical reactions	Chest wall ulcer	M	Not reported	None	Recovered
**4.**	Sharma and Kalawat, 2010	35/F	-	-	Infected burns	Not reported/ Biochemical reactions	Right leg burns	M	Not reported	None	Recovered

**5.**	Prinja et al., 2013	92/M	Sea water exposure	DM	Cellulitis and wound infection	Wound swabs/ Not reported	Open fracture of the calcaneum/ ankle after boat accident	M	Not reported	Amoclav; Peni + Fluclox; Cipro	Recovered
**6.**	Yohe et al., 1997	61/M	Place of residence, PAVD	COPD, PAVD, vascular insufficiency of the lower legs	Abscess of the foot	Abscess drainage/ Biochemical reactions and VITEK System (bioMérieux Vitek, Inc., Hazelwood, Mo.)	Not reported	P	No	Ceftr + Clinda; TMP/SMX	Recovered
**7.**	Otsuka et al., 2007	67/M	HBD, consumption of raw fish	primary sclerosing cholangitis with decompensated liver cirrhosis, distal GE for early gastric cancer	Cellulitis and septicemia	Blood cultures/ Automated and semiautomated analyzers (VITEK2 and API 20NE; BioMerieux, Tokyo, Japan)	Not reported	M	Yes	Imi; Cipro	Died (MOF)
**8.**	Chen et al., 1997	73/M	HBD	Alcoholism, fatty liver	Spontaneous periorbitofacial cellulitis with sepsis	Blood culture, facial tissue/ ID 32 GN profile (API system; bioMérieux, Marcy l'Etoile, France)	Not reported	M	Yes	Ctax + Clinda + Genta	Died
**9.**	Chen et al., 1997	53/M	Malignancy, cachexia	Terminal rectal carcinoma with intraabdominal carcinomatosis	Perineal abscess with numerous peritoneocutaneous fistulas	Pus/ ID 32 GN profile (API system; bioMérieux, Marcy l'Etoile, France)	Not reported	M	No	Cefa + Genta	Died because of cachexia due to underlying malignancy
**10.**	Chen et al., 1997	69/M	HBD	Cholelithiasis, chronic cholezystitis	Cellulitis, postcholecystectomy wound infection, subcutaneous abscess	Pus/ ID 32 GN profile (API system; bioMérieux, Marcy l'Etoile, France)	Not reported	P	Not reported	Cefa + Genta; Clex	Recovered

**11.**	Chen et al., 1997	53/F	Immuno-suppression	Aplastic anaemia	Finger cellulitis	Pus/ ID 32 GN profile (API system; bioMérieux, Marcy l'Etoile, France)	Fish punctured wound	P	Not reported	Cefa + Genta; Pip + Genta + Vanco	Recovered
**12.**	Ryan et al., 2018	40/F	Exposure to saltwater flora	-	Soft tissue infection of the left palm	Culture from the wound/ Not reported	Hand laceration, retained seashall fragments	M	No	Pip/Taz + Vanco + Cipro; Cipro + Doxy	Recovered
**13.**	Giroux et al., 2017	67/M	Fishing, DM	DM	Necrotizing fascitis of the lower limb	Blood cultures, tissue/ Bruker MALDI-TOF Mass Spectrometry	Not reported	P	Yes	Amoclav; Pip/Taz + Clinda + Amika; Pip/Taz + Clinda; Ctax + Line + Metro	Recovered
**14.**	Tang et al., 2016	65/M	Renal disease, DM	Hypertension, DM, hyperlipidemia, nephrotic syndrome, chronic renal impairment, congestive heart failure	Fournier´s gangrene with sepsis, DIC, ACS	Scotal fluid/ Vitek 2, subsequent MALDI-TOF	Not reported	P	No	Amoclav; Cefta	Died
**15.**	Patel et al., 2020	38/M	Fish tank and puffer fish exposure	Prior left thumb dislocation, bilateral carpal tunnel releases, traumatic brain injury, depression, anxiety, migraines, fibromyalgia, arthritis, smoker	Flexor tenosynovitis	Distal tendon sheat cultures/ Not reported	Penetrating injury via new, clean drill bit with further trauma	P	No	Vanco + Pip/Taz; TMP/SMX + Cipro; TMP/SMX	Recovered, still limited due to stiffness

**16.**	Mohr et al., 2016	53/M	Immuno-suppression, ESRD, DM	ESRD, DM, heart-Tx, pacemaker, CMV colitis	Soft tissue infection of the left foot	Wound swab, tissue biopsies/ Not reported	Foot ulcer	P	No	Mero + Vanco	Recovered
**17.**	Latif et al., 2019	70/M	HBD	CAD, alcoholic liver cirrhosis with TIPSS	Sepsis secondary to cellutitis	Blood cultures/ VITEK (bioMerieux)	Chronic stasis dermatitis	M	Yes	Vanco + Mero; Levo	Recovered
**18.**	Pagani et al., 2003	87/M	Immuno-suppression, sea water exposure	Rheumatic myalgia with long-term, low-dose methyl-prednisolone, obesity	Erysipelas of the left forearm and pneumonic infiltrates	Blood cultures/ VITEK II automated system (bioMérieux, Marcy l'Etoile, France), semiautomated Api ID 32 GN (bioMérieux)	Cutaneous-subcutaneous wound left elbow	M	Yes	Ampi/Sulb; Ampi	Recovered
**19.**	Brink et al., 1995	36/ Not reported	Poor living standards	Not reported	Cellulitis	Blood cultures/ Biochemical reactions and API 20NE system (bioMérieux, Marcy l'Etoile, France)	Traumatic ulcer on lower extremity	P	Yes	Not reported	Recovered
**Arthritis and osteomyelitis**											
**20.**	Levy and Tessier, 1998	48/M	Sea water exposure	-	Arthritis (proximal interphalangeal articulation of the left foot) with cellulitis and fistula	Articular fluid, periarticular tissue/ API 20NE System (bioMérieux, Marcy-I'Etoile, France)	Puncture from sea urchins needle	M	No blood cultures performed	Cipro; Ceftr	Persistent edema and fistula 2 weeks after end of treatment

**21.**	Carlson and Dux, 2013	77/M	PAVD, chronic lower extremity ulceration, poor hygiene	Pancytopenia, PAVD, hypertension, hyperlipidemia, renal failure	Bilateral calcaneal osteomyelitis	Bone biopsy/ Microscan Autoscan 4 System (Siemens Healthcare Diagnostics)	Bilateral heel ulcerations	M	No	Pip/Taz + Vanco	Clinical Improvement
**22.**	Guinetti-Ortiz et al., 2016	48/M	Place of residence	Parkinson disease	Osteomyelitis of the right metatarsal bone	Bone biopsy/ Automated Vitek-2	Not reported	M	No	Vanco + Imi; Pip/Taz, Amoclav + Cipro	Clinical improvement
**Eye infections**											
**23.**	Butt et al., 1997	37/M	Sea water exposure	-	Severe suppurative eye infection with persistent endophthalmitis	Vitreous fluid/ Not reported	Eye injury by a fishhook while fishing	P	Not reported	Genta, Cefa; Pip/Taz+Genta; Cipro	Survived, Enucleation
**24.**	Mohan et al., 2014	25/M	Fisherman	-	Post-traumatic endophthalmitis	Vitreous sample/ Vitek 2 (BioMerieux, Marcy l'Etoile, France)	Open globe fishhook injury	M	No	Vanco + Cefta, Gati	Clinical improvement
**Infections associated with dialysis**											
**25.**	Bhandari et al., 2020	69/M	River water exposure, ESRD/ CAPD, DM, HBD	Polycystic kidney disease with CAPD, polycystic liver, coronary artery bypass, DM, CCE	CAPD peritonitis associated with complex, multi-loculated splenic abscess	Peritoneal fluid, aspirate of abcess-drainage, blood cultures/ Not reported	Lower leg ulcer or colonization of the PD exite site	P	Yes	Initially broad-spectrum antibiotics; Cipro + Metro	Survived, recommenced CAPD without complication

**26.**	Yim et al., 2010	67/M	Place of residence, ESRD/ CAPD	Chronic glomerulo-nephritis, CAPD-peritonitis in the past	Severe sepsis due to CAPD peritonitis, necrotizing fasciitis	Blood cultures, peritoneal fluid/ Not reported	Left flank ulcer	M	Yes	Cefta + Mero + Vanco + Doxy; Genta + Cefta	Died (MOF)
**27.**	Shrishrimal, 2012	78/M	ESRD/ dialysis, PAVD, DM	DM, PAVD, multiple amputations, chronic dialysis via a tunneled hemodialysis catheter	Catheter-associated blood stream infection	Blood cultures, tip of the hemodialysis catheter/ Not reported	Not reported	M	Yes	Vanco + Cefe; Genta; Genta + Cipro; Mero	Recovered
**28.**	Chang et al., 2005	54/F	Malignancy, ESRD/ CAPD	Chronic glomerulo-nephritis with CAPD, colon cancer with liver metastasis	CAPD-associated Peritonitis	Dialysate/ Not reported	Gut translocation	P	No	Cefa + Tobra	Clinical improvement
**29.**	Vickers and Ullian, 2011	42/F	ESRD/ PD, frequently fishing, place of residence, DM	ESRD with PD by diabetic glomerulo-sclerosis, DM	Recurrent peritonitis associated with PD	PD fluid/ Not reported	Not reported	M	Not reported	Cefa + Genta; Genta; Cipro + Genta	Survived, switched to hemodialysis
**30.**	López Aperador et al., 2016	40/F	ESRD/ PD, exposure to sea water	Takayasu-Arteritis, ESRD with PD	Peritonitis associated with PD	Peritoneal fluid/ Not reported	PD-catheter	P	No	Cefta + Vanco; Cefta + Cipro + Fluco	Recovered
**31.**	Chen et al., 1997	57/M	Dialysis	Glucophosphate intoxication, acute renal failure	Peritonitis	Dialysate/ ID 32 GN profile (API system; bioMérieux, Marcy l'Etoile, France)	Not reported	P	No	Cefta	Recovered

**32.**	Lee et al., 2016	82/F	ESRD/ dialysis, malignancy	ESRD, cervical cancer with bladder invasion complicating by bilateral hydronephrosis, complicated urinary tract infection	Febrile (maybe catheter-related) infection	Blood cultures (peripheral vessels + intrapermanent catheter)/ Phoenix NID card (Becton Dickinson Diagnostic Systems, Sparks, MD, USA), MALDI-TOF (Bruker Daltonik GmbH, Bremen, Germany)	Catheter-related	M	Yes	Dori	Recovered
**Cerebral infections**											
**33.**	Süzüku et al., 2004	9/F	Place of residence	Otitis media 2 years ago, intermittant otorrhoea	Chronic otitis media with cerebellar abscess	Aspirate of the cerebellar abscess/ API 20E system (bioMeriux, France) and VITEK system (bioMeriux, France)	Chronic otitis media	M	Not reported	Ampi/Sulb	Recovered
**34.**	Yilmaz et al., 2007	28/M	Poor personal hygiene, trap fisherman, river water exposure	Chronic suppurative otitis media	Cerebral abscess and meningitis	CSF, ear exsudate/ Phoenix system (Becton Dickinson)	Chronic otitis media with mastoiditis	P	No	Mero	Recovered
**35.**	Duan et al., 2015	63/F	-	-	Intracranial infection after surgery of cerebral hemorrhage	CSF/ 16S rDNA amplification assay	Not reported	M	No	Tica/Clav + Imi	Recovered
**Infective endocarditis**											
**36.**	Dhawan et al., 1998	24/F	-	Rheumatic heart disease	Infective endocarditis	Blood cultures/ Biochemical reactions and API 20E system (bioMerieux Vitek, Inc., Hazelwood, Mo.)	Not reported	P	Yes	Genta + Peni	Clinical improvement

**37.**	Constant et al., 2014	40/M	-	Seizure disorder, dislipidemia, tobacco use, asthma, bronchitis, sleep apnea, hypertension, hypothyroidism, osteoarthritis, reight carpal tunnel release surgery, venous insufficiency, thrombosis of the right radial vein	Infective endocarditis	Blood cultures/ Vitek 2 automated system (bioMérieux, Durham, NC)	Burns wound	M	Yes	Cefa; Cefe + Genta	Lost to FU
**Intra-abdominal infections**											
**38.**	Wang et al., 2004	61/F	HBD	CCE	Febrile diarrhea with polymicrobial bacteremia	Blood cultures/ Biochemical reactions and semiautomated ATB ID 32 GN method (bioMérieux)	Shark meat consumption	P	Yes	Cefa + Genta; Cefix + Doxy	Recovered
**39.**	Muñoz et al., 2015	60/M	HBD, malnutrition, consumption of crustaceans	Choledocho-lithiasis with biliary pancreatitis, CCE	Recurrent pancreatitis with infected pseudocyst	Fluid of infected pancreatic pseudocyst/ Not reported	Not reported	P	Not reported	Mero	Not reported
**40.**	Baruah and Grover, 2014	50/M	HBD	Alcoholic liver cirrhosis, chronic bidi smoker, alcoholism	Peritonitis/ ascitic fluid infection	Ascitic fluid/ Automated Vitek-2 Compact(C) system version 6.01 (Biomerieux, North Carolina/USA)	Introducing by repeated ascitic tapping	M	No	Pip/Taz	Recovered
**41.**	Basir et al., 2012	22/F	DM	DM	Splenic abscess, pneumonia, septic shock	Blood cultures/ API 20NE (Biomerieux, Marcy L'Etoile, France)	Not reported	M	Yes	Cefu + Azithro; Mero; Cefta + Amoclav; Cipro	Recovered

**42.**	Chen et al., 1997	83/M	HBD, Malignancy	Biliary tract lithiasis, prostate carcinoma	Biliary tract infection with hepatic abscess	Bile/ ID 32 GN profile (API system; bioMérieux, Marcy l'Etoile, France)	Not reported	P	No	Cefa + Genta + Metro, Cper + Clinda	Recovered
**43.**	Chen et al., 1997	52/M	HBD	Biliary tract lithiasis	Biliary tract infection with hepatic abscess	Bile/ ID 32 GN profile (API system; bioMérieux, Marcy l'Etoile, France)	Not reported	P	No	Cefa + Genta + Metro	Recovered
**44.**	Chen et al., 1997	71/M	HBD	Biliary tract lithiasis	Biliary tract infection	Bile/ ID 32 GN profile (API system; bioMérieux, Marcy l'Etoile, France)	Not reported	P	No	Cefa + Genta	Recovered
**45.**	Chen et al., 1997	81/M	HBD	Biliary tract lithiasis	Biliary tract infection	Bile, Blood cultures/ ID 32 GN profile (API system; bioMérieux, Marcy l'Etoile, France)	Not reported	P	Yes	Cefa + Genta + Metro	Recovered
**46.**	Bulat et al., 2018	27/M	-	Victim of aggressive blunt trauma with perforation on the ileum	Septic shock with MODS bei general purulent peritonitis and multiple intraperitoneal abscesses	Peritoneal fluid/ Not reported	GUT perforation	P	No	Erta + Vanco	Recovered
**47.**	Ali et al., 2017	70/M	-	Chronic constipation, hemorrhoids	Subacute intestinal obstruction and appendidicitis	Peritoneal fluid/ API® 20E (Biomérieux, Marcy l'Etoile, France)	Not reported	M	No	Metro + Pip/Taz; Metro + Cipro; Tz + Levo	Recovered
**Respiratory tract infections**											
**48.**	Durdu et al., 2012	43/F	-	Brochiectasis, recurrent pneumonias	Pneumonia	Sputum/ API 20 NE (BioMerieux, France)	Not reported	M	No	Cipro + Cefta; Ceftr	Recovered

**49.**	Jorens et al., 2004	65/M	DM	Severe polytrauma with pneumothorax, anterior myocardial infarction with cardiogenic shock, CAD, congestive heart failure, DM	Ventilator-associated pneumonia	Bronchus aspirates, BAL/ Not reported	Nasal cavity	P	No	Amoclav	Recovered
**50.**	Patel et al., 2012	63/M	Sea water exposure	NSTEMI, resuscitation	Pneumonia	Endotracheal aspirate/ API 20 NE (BioMerieux)	Respiratory tract	M	No	Vanco + Pip/Taz; Cefe	Recovered
**51.**	Liao et al., 2000	58/M	Malignancy	Cavitating, squamous cell carcinoma oft he lung	Infected cavitating lung tumor	Aspirate of cavitating lung tumors/ Not reported	Not reported	P	Not reported	Not reported	Clinical improvement
**Bloodstream infections**											
**52.**	Chen et al., 1997	51/M	HBD	Biliary tract lithiasis, chronic hepatitis B	Septicemia	Blood cultures/ ID 32 GN profile (API system; bioMérieux, Marcy l'Etoile, France)	Not reported	P	Yes	Cefa + Genta + Metro	Recovered
**53.**	Chen et al., 1997	71/M	HBD	Biliary tract lithiasis	Septicemia	Blood cultures/ ID 32 GN profile (API system; bioMérieux, Marcy l'Etoile, France)	Not reported	P	Yes	Cefa + Genta; Cper	Recovered
**54.**	Chen et al., 1997	61/M	HBD	Liver cirrhosis with esophageal varices and gastric varices on endoscopic injection sclerosing therapy	Septicemia	Blood cultures/ ID 32 GN profile (API system; bioMérieux, Marcy l'Etoile, France)	Not reported	P	Yes	Cefa + Genta	Recovered

**55.**	Chen et al., 1997	71/F	Malignancy	Cholangio-carcinoma with liver invasion	Septicemia	Blood cultures/ ID 32 GN profile (API system; bioMérieux, Marcy l'Etoile, France)	Not reported	P	Yes	Cefa + Genta	Died
**56.**	Chen et al., 1997	85/M	Malignancy	Pancreatic carcinoma with liver metastasis	Septicemia	Blood cultures/ ID 32 GN profile (API system; bioMérieux, Marcy l'Etoile, France)	Not reported	P	Yes	Cefa + Genta	Died
**57.**	Ranjan and Chowdhary, 2017	24/M	-	-	Bacteriemia after road traffic accident with head injury and polytrauma	Blood cultures/ Vitek® 2 system (bioMerieux Inc., 100 Rodlphe Street, Durham, NC, USA)	Not reported	M	Yes	Pip/Taz + Genta	Died
**58.**	Brink et al., 1995	18/ Not reported	Poor living standards	Not reported	Sepsis	Blood cultures/ Biochemical reactions and API 20NE system (bioMérieux, Marcy l'Etoile, France)	Burn wounds 35% of the body	P	Yes	Not reported	Recovered
**59.**	Brink et al., 1995	18/ Not reported	Poor living standards	Adult respiratory distress syndrom	Sepsis	Blood cultures/ Biochemical reactions and API 20NE system (bioMérieux, Marcy l'Etoile, France)	Burn wounds 50% of the body	P	Yes	Not reported	Died
**60.**	Brink et al., 1995	57/ Not reported	Poor living standards	Tetanus	Sepsis	Blood cultures/ Biochemical reactions and API 20NE system (bioMérieux, Marcy l'Etoile, France)	Traumatic ulcer on lower extremity	M	Yes	Not reported	Recovered
**61.**	Brink et al., 1995	75/ Not reported	Poor living standards, DM	DM	Bacteremia	Blood cultures/ Biochemical reactions and API 20NE system (bioMérieux, Marcy l'Etoile, France)	Diabetic ulcer on lower extremity	M	Yes	Not reported	Recovered

**62.**	Brink et al., 1995	21/ Not reported	Poor living standards	Chemical burnwounds on esophagus	Sepsis	Blood cultures/ Biochemical reactions and API 20NE system (bioMérieux, Marcy l'Etoile, France)	Not reported	P	Yes	Not reported	Recovered
**63.**	Brink et al., 1995	52/ Not reported	Poor living standards	Epilepsy, aspiration	Bacteremia	Blood cultures/ Biochemical reactions and API 20NE system (bioMérieux, Marcy l'Etoile, France)	Not reported	M	Yes	Not reported	Recovered
**Pediatric infections**											
**64.**	Brink et al., 1995	1 day/ Not reported	Poor living standards, LBW	Respiratory distress, patent ductus arteriosus	Sepsis	Blood cultures/ Biochemical reactions and API 20NE system (bioMérieux, Marcy l'Etoile, France)	Not reported	P	Yes	Not reported	Died
**65.**	Brink et al., 1995	1 day/ Not reported	Poor living standards, LBW	Respiratory distress, asphyxia neonatorum	Sepsis	Blood cultures/ Biochemical reactions and API 20NE system (bioMérieux, Marcy l'Etoile, France)	Not reported	M	Yes	Not reported	Died
**66-68.**	Brink et al., 1995	1 day/ Not reported	Poor living standards, LBW	Respiratory distress, pneumonia	Bacteremia	Blood cultures/ Biochemical reactions and API 20NE system (bioMérieux, Marcy l'Etoile, France)	Not reported	P	Yes	Not reported	Recovered
**69.**	Brink et al., 1995	1 day/ Not reported	Poor living standards, LBW	Respiratory distress, Bochdalek´s hernia	Sepsis	Blood cultures/ Biochemical reactions and API 20NE system (bioMérieux, Marcy l'Etoile, France)	Not reported	P	Yes	Not reported	Died

**70-71.**	Brink et al., 1995	1 day/ Not reported	Poor living standards, LBW	Respiratory distress, meconium aspiration	Bacteremia	Blood cultures/ Biochemical reactions and API 20NE system (bioMérieux, Marcy l'Etoile, France)	Not reported	P	Yes	Not reported	Recovered
**72.**	Brink et al., 1995	Prema-ture/ Not reported	Poor living standards, LBW	Respiratory distress, congenital syphilis	Sepsis	Blood cultures/ Biochemical reactions and API 20NE system (bioMérieux, Marcy l'Etoile, France)	Not reported	M	Yes	Not reported	Died
**73.**	Brink et al., 1995	Prema-ture/ Not reported	Poor living standards, LBW	Respiratory distress, pneumonia	Sepsis	Blood cultures/ Biochemical reactions and API 20NE system (bioMérieux, Marcy l'Etoile, France)	Not reported	P	Yes	Not reported	Died
**74.**	Brink et al., 1995	Prema-ture/ Not reported	Poor living standards, LBW	Respiratory distress, congenital syphilis	Sepsis	Blood cultures/ Biochemical reactions and API 20NE system (bioMérieux, Marcy l'Etoile, France)	Not reported	P	Yes	Not reported	Died
**75.**	Brink et al., 1995	Prema-ture/ Not reported	Poor living standards, LBW	Respiratory distress	Bacteremia	Blood cultures/ Biochemical reactions and API 20NE system (bioMérieux, Marcy l'Etoile, France)	Not reported	P	Yes	Not reported	Recovered
**76.**	Brink et al., 1995	Prema-ture/ Not reported	Poor living standards, LBW	Respiratory distress, hydrocephalus	Bacteremia	Blood cultures/ Biochemical reactions and API 20NE system (bioMérieux, Marcy l'Etoile, France)	Not reported	P	Yes	Not reported	Recovered
**77-78.**	Brink et al., 1995	Prema-ture/ Not reported	Poor living standards, LBW	Respiratory distress	Bacteremia	Blood cultures/ Biochemical reactions and API 20NE system (bioMérieux, Marcy l'Etoile, France)	Not reported	M	Yes	Not reported	Recovered

**79.**	Brink et al., 1995	Prema-ture/ Not reported	Poor living standards, LBW	Respiratory distress, congenital laryngeal cyst	Bacteremia	Blood cultures/ Biochemical reactions and API 20NE system (bioMérieux, Marcy l'Etoile, France)	Not reported	M	Yes	Not reported	Recovered
**80.**	Brink et al., 1995	6 months/ Not reported	Poor living standards, LBW	Recurrent broncho-pneumonia	Sepsis	Blood cultures/ Biochemical reactions and API 20NE system (bioMérieux, Marcy l'Etoile, France)	Not reported	M	Yes	Not reported	Recovered
**81.**	Brink et al., 1995	12 months/ Not reported	Poor living standards, LBW	Broncho-pneumonia	Bacteremia	Blood cultures/ Biochemical reactions and API 20NE system (bioMérieux, Marcy l'Etoile, France)	Not reported	P	Yes	Not reported	Recovered
**82.**	Brink et al., 1995	12 months/ Not reported	Poor living standards, LBW	Broncho-pneumonia	Bacteremia	Blood cultures/ Biochemical reactions and API 20NE system (bioMérieux, Marcy l'Etoile, France)	Not reported	M	Yes	Not reported	Recovered
**83.**	Brink et al., 1995	16/ Not reported	Poor living standards	Not reported	Sepsis	Blood cultures/ Biochemical reactions and API 20NE system (bioMérieux, Marcy l'Etoile, France)	Burn wounds 35% of the body	P	Yes	Not reported	Recovered
**84.**	Brink et al., 1995	17/F	Poor living standards, HBD	Cholangitis, liver abscess, liver failure	Sepsis	Blood cultures/ Biochemical reactions and API 20NE system (bioMérieux, Marcy l'Etoile, France)	Not reported	P	Yes	Not reported	Died

**Others**											
**85.**	Benaissa et al., 2021	66/M	-	Hypertension, benign prostate hypertrophy, smoking, CAD/ myocardial infarction	Septic shock and retroperitoneal hematoma secondary to coronary angiography	Blood cultures/ API 20NE® gallery (Bio- Mérieux, Marcy l′étoile France) and biochemical and bacteriological tests	Catheter used for coronary angiography	M	Yes	Ceftr + Cipro; Tige + Amika	Recovered
**86.**	Chen et al., 1997	71/M	-	Obstructed nasolacrimal duct	Dacryocystitis	Pus/ ID 32 GN profile (API system; bioMérieux, Marcy l'Etoile, France)	Not reported	P	No	Clex	Recovered
**87.**	Chen et al., 1997	63/M	HBD	Biliary tract lithiasis; biliary tract infection with pancreatic abszess, pneumonia, schizophrenia	Pleural empyema, sepsis	Pleural effusion/ ID 32 GN profile (API system; bioMérieux, Marcy l'Etoile, France)	Not reported	P	No	Cefa + Genta; Cper + Genta	Died

Vanco= Vancomycin, Imi= Imipenem, Pip/Taz= Piperacillin/Tazobactam, Amoclav= Amoxicillin/Clavulanic acid, Clav= Clavulanic acid, Cipro= Ciprofloxacin, Cefa= Cefazoline, Ceftr= Ceftriaxone, Ampi/Sulb= Ampicillin/Sulbactam, Metro= Metronidazole, Ampi= Ampicillin, Peni= Penicillin, Cefix= Cefixime, Mero= Meropenem, Cefta= Ceftazidime, Fluclox= Flucloxacillin, Tobra= Tobramycin, Cefe= Cefepime, Fluco= Fluconazole, Cefu= Cefuroxime, Azithro= Azithromycin, Tige= Tigecyclin, Amika= Amikacin, Gati= Gatifloxacin, Levo= Levofloxacin, Pip= Piperacillin, Clex= Cephalexin, Cper= Cefoperazone, Erta= Ertapeneme, Ctax= Cefotaxime, Dori= Doripenem, Tz= Tazozin, Tica= Ticarcillin, Line= Linezolid

PD= Peritoneal dialysis, DM= Diabetes mellitus, CAPD= Continuous ambulatory peritoneal dialysis, CCE= Cholezystectomy, MOF= Multi-organ-failure, ESRD= End-stage renal disease, BAL= Bronchoalveolar lavage, FU= Follow up, HBD= Hepato-biliary disorder, COPD= Chronic obstructive pulmonary disease, Tx= Transplantation, CMV= Cytomegalovirus CSF= Cerebrospinal fluid, CAD= Coronary artery disease, MODS= Multi-organ-dysfunction-syndrom, GE= Gastrectomy, ACS= Acute coronary syndrom, DIC= Disseminated intravascular coagulation, LBW= Low birth weight

Ali, A. M., Noorulamin, M., and Arif, S. (2017). Isolation of *Shewanella putrefaciens* in an elderly man with subacute intestinal obstruction & appendicitis. *IDCases*. 9, 45–46. doi: 10.1016/j.idcr.2017.06.001

Bulat, O., Bulat, C., Blaj, M., Lupusoru, I., and Scripcariu, V. (2018). A Rare Colonization in Peritoneum After Blunt Abdominal Trauma: *S. putrefaciens* and *S. cerevisiae*. *Balkan Med. J*. 35 (4), 333–335. doi: 10.4274/balkanmedj.2017.0773

Jorens, P. G., Goovaerts, K., and Ieven, M. (2004). *Shewanella putrefaciens* isolated in a case of ventilator-associated pneumonia. *Respiration*. 71 (2), 199–201. doi: 10.1159/000076686

Liao, W. Y., Liaw, Y. S., Wang, H. C., Chen, K. Y., Luh, K. T., and Yang, P. C. (2000). Bacteriology of infected cavitating lung tumor. *Am. J. Respir. Crit. Care Med*. 161 (5), 1750–1753. doi: 10.1164/ajrccm.161.5.9905103

Ranjan, R., and Chowdhary, P. (2017). A rare case of *Shewanella putrefaciens* bacteremia in a patient of road traffic accident. *Indian J. Pathol. Microbiol*. 60 (4), 599–600. doi: 10.4103/IJPM.IJPM_254_16

Wang, I. K., Lee, M. H., Chen, Y. M., and Huang, C. C. (2004). Polymicrobial bacteremia caused by Escherichia Coli, Edwardsiella Tarda, and Shewanella putrefaciens. *Chang Gung Med. J*. 27 (9), 701–705.
